# Feature-Based Fusion of Angiography Videos and Blood Parameters for Fractional Flow Reserve (FFR) Prediction

**DOI:** 10.3390/diagnostics16213496

**Published:** 2026-10-26

**Authors:** Mehmet Nazir Kaçar, Çağrı Yayla, Dilek Kaçar, İlkay Ulusoy

**Affiliations:** 1 Department of Electrical and Electronics Engineering, Middle East Technical University, 06800 Ankara, Türkiye; ilkay@metu.edu.tr; 2 Department of Cardiology, Ankara Bilkent City Hospital, University of Health Sciences, 06800 Ankara, Türkiye; cagriyayla@gmail.com; 3 Department of Pediatric Hematology Oncology, Ankara Bilkent City Hospital, University of Health Sciences, 06800 Ankara, Türkiye; dilekbank@yahoo.com

**Keywords:** angiography, machine learning, complete blood count, coronary artery disease, fractional flow reserve

## Abstract

**Background/Objectives:** Estimating fractional flow reserve (FFR) non-invasively in intermediate coronary artery disease remains challenging because anatomical stenosis severity does not consistently correlate with functional ischemia. While conventional image-based models typically assume simplified fluid dynamics, functional flow may also be influenced by blood cellular properties. We evaluated a multimodal neural network framework that conditions angiographic representations on hemorheological surrogate parameters derived directly from routine complete blood count (CBC) data. **Methods:** Retrospective data from 179 patients (256 left anterior descending [LAD] videos) with matched invasive FFR were analyzed. To incorporate cellular flow tendencies without specialized assays, routine CBC parameters were configured into a 5-parameter Blood Fluidity Model (BFM) comprising gender, baseline mean platelet volume (MPV), and three engineered surrogate metrics: Erythrocyte Fragility Index (red blood cell distribution width [RDW] × mean corpuscular hemoglobin concentration [MCHC]), Cell-Free Hemoglobin Index (hemoglobin [HGB]—(hematocrit [HCT]/3)), and Erythrocyte-Platelet Volume Balance (HCT/plateletcrit [PCT]). A Feature-wise Linear Modulation (FiLM) architecture was used to modulate three-dimensional convolutional neural network (3D-CNN) spatiotemporal features extracted from angiography videos. Evaluation relied on patient-grouped 5-fold cross-validation and an independent test set (36 patients). **Results:** On the independent test set, the video-only baseline showed limited predictive utility (area under curve [AUC]: 0.482). The multimodal (angiography video features modulated by BFM) FiLM model improved upon angiography video-only, BFM-only (AUC: 0.599), and video late-fusion with BFM baselines (AUC: 0.521), achieving an AUC of 0.621, a balanced accuracy of 0.590, and a statistically significant correlation with invasive FFR (*r_s_* = 0.246, *p* = 0.003). Engineered hemorheological surrogates outperformed raw hematological inputs (all blood parameters only: AUC: 0.601. Multimodal Late Fusion AUC: 0.478. Multimodal FiLM AUC: 0.570). **Conclusions:** Integrating novel CBC-derived biophysical parameters alongside angiographic sequences demonstrates the feasibility of combining blood rheology and vessel geometry into a single computational framework. While observed performance gains remain modest, this approach illustrates the potential of leveraging routine laboratory data to support non-invasive physiological evaluation without incurring additional procedural burden.

## 1. Introduction

The management of coronary artery disease (CAD) has shifted from an anatomy-based approach to a physiology-guided paradigm, transforming clinical decision-making. Fractional Flow Reserve (FFR) remains the primary clinical reference for assessing the functional significance of epicardial coronary stenoses, with robust evidence supporting its use to optimize interventional strategies and improve outcomes [1,2]. To reduce financial costs, technical complexity, and pharmacological risks of traditional pressure-wire-based measurements, non-invasive, angiography-derived FFR algorithms have emerged. Initially, these platforms relied on physics-based computational fluid dynamics (CFD) applied to 3D vessel reconstructions. However, conventional 3D-CFD is computationally intensive and struggles to account for vascular distensibility and microcirculatory resistance mechanisms [3]. To enable real-time intraoperative use, contemporary research has shifted toward deep learning architectures, using convolutional networks and spatio-temporal transformers, to estimate FFR directly from invasive coronary angiography (ICA) [4,5].

However, luminal geometry alone poorly reflects functional ischemia. In the landmark FAME study, up to 65% of intermediate and 20% of severe stenoses were functionally non-ischemic [6]. From a fluid-dynamics perspective, this discrepancy arises from Poiseuille’s law, which states that the pressure gradient across a constriction is proportional to the fluid’s viscosity [7]. In coronary hemodynamics, Demkin et al. demonstrated through Navier–Stokes simulations that post-stenotic pressure loss is highly sensitive to blood viscosity; borderline stenoses that appear non-ischemic under normal fluid viscosity become hemodynamically significant under hyperviscous conditions [8]. Clinically, elevated whole blood viscosity (WBV) calculated via De Simone’s formula is associated with wire-based functional ischemia [9,10] and strongly correlates with systemic endothelial inflammation [11]. Nevertheless, formula-derived viscosity alone cannot fully capture intra-coronary cellular mechanics because patient-specific variations in erythrocyte deformability, cell partitioning, and local spatial distribution under pathological conditions directly determine vascular resistance and wall shear stress [12,13]. Treating blood as an idealized Newtonian liquid oversimplifies these cellular interaction dynamics, creating a diagnostic gap in geometry-only FFR estimation platforms.

To bypass 3D-CFD latency while capturing functional flow, deep learning frameworks for ICA-derived FFR have evolved along two primary trajectories: angiography video-only processing and multimodal feature fusion. Angiography video-only architectures, such as those proposed by Mineo et al., have reported high diagnostic accuracy by capturing dynamic contrast wash-in/wash-out kinetics directly from raw sequences [5]. However, full-frame video processing inherently incurs massive computational demands and remains highly sensitive to background X-ray noise, structural overlap, and motion artifacts. Conversely, region-of-interest (ROI) cropping offers a computationally lightweight alternative focused on target lesion anatomy for real-time inference, but unconditioned video-only baselines often struggle to decouple complex flow kinetics from background noise [4]. Recognizing these limitations, multimodal approaches have incorporated patient-specific variables. For instance, Cho et al. integrated clinical metrics with stenosis features to achieve a high diagnostic performance [14]. Similarly, physics-informed neural networks (PINN) and quantitative flow ratio (QFR) platforms have incorporated pressure-gradient boundary conditions [15,16]. Yet these methodologies predominantly rely on manual quantitative coronary angiography (QCA) feature extraction or assume homogeneous fluid behavior, omitting the physical contributions of cellular hemorheology.

To bridge this physiological and computational gap, integrating macrovascular imaging with patient-specific micro-hemorheology offers a promising, novel paradigm for non-invasive FFR prediction. In this preliminary study, we introduce a diagnostic framework that conditions localized ICA angiographic video crops with a patient-specific Blood Fluidity Model (BFM) derived from routine pre-procedural complete blood count (CBC) parameters. By leveraging baseline erythrocyte and platelet metrics as surrogate markers for underlying cellular hemorheology, our approach seeks to convert routine hematological data into a biologically informed, supplementary predictive signal. Technically, rather than relying on manual QCA segmentations or computationally heavy 3D-CFD simulations, we explore Feature-wise Linear Modulation (FiLM) to dynamically scale and shift deep visual feature maps extracted from ICA video crops based on the patient’s BFM profile. A fundamental model for video feature extraction, a three-dimensional convolutional neural network (3D-CNN), is used for feature extraction from angiography videos. This feature-level fusion is an initial step toward evaluating target stenosis geometry in interaction with non-Newtonian blood fluidity, offering a lightweight, automated framework to better understand the biomechanical interplay between vascular morphology and cellular blood rheology.

## 2. Materials and Methods

### 2.1. Patient Cohort, Imaging Modalities, and Laboratory Assays

1255 patients underwent invasive pressure-wire FFR for suspected or documented CAD at Ankara Bilkent City Hospital between 1 February 2019 and 1 October 2023, with 16,920 angiographic videos. Invasive FFR measurements were performed in accordance with current clinical practice guidelines. A standard 0.014-inch pressure wire (e.g., PressureWire X, Abbott Vascular, Santa Clara, CA, USA/PrimeWire Prestige, Volcano Corporation/Philips, San Diego, CA, USA/OmniWire pressure guide wire, Philips Image Guided Therapy Devices, San Diego, CA, USA) was zeroed and equalized with aortic pressure at the tip of the guiding catheter before crossing the target stenosis. The pressure wire was then advanced into the distal third of the target vessel, placing the pressure sensor at least 2–3 cm distal to the stenosis. Maximal hyperemia was induced either via continuous intravenous infusion of adenosine (140 μg/kg/min) or intracoronary bolus injections (60–120 μg). FFR was calculated as the ratio of mean distal coronary pressure (P_d_) to mean aortic pressure (P_a_) during peak stable hyperemia (P_d_/P_a_). An FFR value of ≤0.80 was defined as the binary cutoff for hemodynamically significant ischemia.

**Figure 1:**
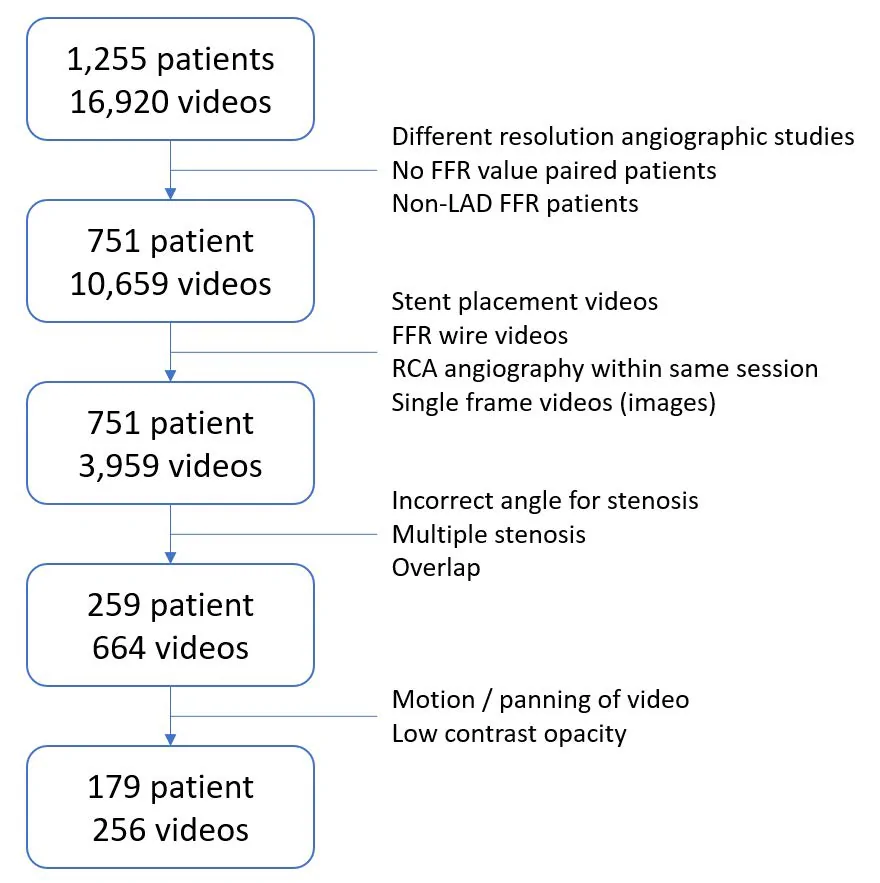
Study flow diagram.

Patients were excluded if FFR couldn’t be synchronized with angiographic videos. Non-LAD interventions were excluded for anatomical consistency, leaving 751 patients evaluated for LAD stenoses. Further screening removed images with low contrast, artifacts, vessel overlaps, previous stenting, or catheter interference. The final dataset included 256 high-quality, single-stenosis angiographic videos from 179 patients, each linked to its FFR value. FFR values were centered around the 0.80 cutoff. A detailed study flow diagram is given in Figure 1.

Invasive coronary angiograms were acquired using standard protocols (GE Innova Digital Angiography platform). Native DICOM sequences (1000 × 1000 pixels) were preprocessed by discarding initial and terminal frames. Dynamic sequences were standardized to 32 frames: shorter ones were padded with the final frame, longer ones clipped to the initial 32 frames starting from sequence onset without physiological alignment to contrast arrival or cardiac gating. For spatial preprocessing, an experienced interventional cardiologist manually localized and cropped a 256 × 256 pixel ROI centered on the culprit stenosis. Consequently, this pipeline operates semi-automatically, requiring user input to define the target stenosis ROI before automated downstream feature extraction and FFR conditioning.

Pre-procedural CBC tests conducted within 7 days before angiography were collected as a mandatory inclusion criterion for elective FFR evaluation; no patients were excluded due to missing baseline blood work. When multiple CBC evaluations were available within this 1-week window, we selected the test closest to catheterization. Whole blood samples in EDTA tubes were analyzed with automated systems (ADVIA 2120i). Hematological parameters extracted included erythrocyte and platelet indices such as red blood cell (RBC) count, hemoglobin (Hgb), hematocrit (Hct), mean corpuscular volume (MCV), mean corpuscular hemoglobin concentration (MCHC), red cell distribution width (RDW), platelet count, plateletcrit (PCT), and mean platelet volume (MPV). Gender and age data were also recorded to complete the demographic profile. We linked these parameters to the corresponding angiographic video files using unique identifiers.

### 2.2. Development of the Five-Parameter BFM: Biophysical Rationale and Feature Engineering

#### 2.2.1. Established Biophysical Principles of Intra-Vascular Cellular Flow

In healthy vascular environments, blood exhibits a distinct multicellular organization where large, highly deformable erythrocytes occupy the high-velocity central axial stream. This movement hydrodynamically drives smaller platelets toward the vessel periphery via shear-induced lateral displacement, establishing a pronounced concentration peak near the endothelial boundary [17,18]. Structurally robust platelets with high MPV are efficiently excluded by this central erythrocyte core. Because these larger elements cannot fit into the microscopic interstitial gaps between traveling erythrocytes, they are driven toward the extreme periphery [18]. Crucially, by remaining marginated, these large platelets leave the central erythrocyte core uninhibited, preserving ideal fluidic harmony and maximizing downstream perfusion. Conversely, when platelet volume is depleted (low MPV), smaller platelets escape this geometric exclusion and infiltrate the microgaps within the erythrocyte core. This intra-erythrocytic infiltration directly disrupts the structural and fluidic alignment of traveling erythrocytes, increasing the frequency of chaotic cell-to-cell collisions and amplifying internal viscosity [18]. This spatial disharmony is further compounded by structural vulnerabilities within the erythrocyte compartment itself, characterized by increased erythrocyte size heterogeneity (high RDW) and a concomitant loss of membrane deformability [19,20]. When these rigid erythrocytes encounter a stenotic segment, their altered microfluidic behavior forces them to deviate from their axial trajectory. This axial displacement leads to aberrant margination and direct mechanical friction against the vascular endothelium, thereby promoting downstream microvascular damage [21,22]. Under the turbulent flow within a stenosis, these fragile erythrocytes undergo subclinical micro-fragmentation, releasing free Hgb into the plasma. This cell-free plasma Hgb acts as a high-affinity scavenger of endothelial nitric oxide (NO). This localized NO depletion triggers acute smooth muscle contraction, inducing vasospasms and further restricting downstream absolute blood flow independently of luminal narrowing [23,24].

#### 2.2.2. Feature Engineering Framework and Model Hypotheses

To complement geometry-centric angiography-derived FFR algorithms, we modeled the coronary lumen as a non-Newtonian cellular suspension. The dynamic interplay of circulating blood cells forms the physiological baseline of our BFM. To translate these cellular interactions into a computable predictive model, we extracted and engineered rheological features from standard CBC parameters. We excluded white blood cells because, as nucleated immune cells, their counts fluctuate rapidly during acute inflammatory states. Furthermore, unlike erythrocytes and platelets, routine CBC panels provide only quantitative leukocyte counts and percentages without structural or volumetric indices relevant to micro-rheological modeling. We excluded chronological age to prevent redundancy and collinearity. Although hematological parameters change with age, cellular indices likely measure biological aging. Including both would be redundant; omitting age allows the model to use direct biophysical readouts instead of calendar years. Conversely, we retained gender directly to account for baseline vascular geometry and reference Hct setpoints. We retained baseline MPV as a primary feature to proxy platelet spatial dynamics—specifically, whether larger platelets stay marginated or smaller (low MPV) infiltrate interstitial spaces between erythrocytes, disrupting central flow. In addition to retaining gender and baseline MPV as core features, we engineered three novel surrogate indices from routine CBC parameters to capture distinct biophysical phenomena. To our knowledge, the Erythrocyte Fragility Index (EFI), Cell-Free Hemoglobin Index (CFHI), and Erythrocyte-Platelet Volume Balance (EPVB) are newly formulated parameters proposed for the first time in this study and have not been previously described or validated in the literature.

The first engineered metric, the EFI, was designed to quantify the combined mechanical vulnerability of RBCs, defined as follows:

EFI = RDW (%) × MCHC (g/dL)

While RDW reflects dimensional heterogeneity (anisocytosis) that disrupts geometric alignment within the central axial stream, MCHC serves as a direct proxy for internal cytoplasmic viscosity and membrane rigidity, as higher hemoglobin concentrations restrict cellular deformability. By multiplying these two parameters, EFI captures a synergistic biophysical risk: high EFI denotes an erythrocyte population that is both dimensionally mismatched to streamline flow and internally too rigid to undergo shear-induced deformation. When traversing stenotic segments, high-EFI red cells are susceptible to trajectory deflections, mechanical endothelial friction, and subclinical intravascular hemolysis.

The second metric, CFHI, was engineered to quantify circulating unencapsulated Hgb and subclinical intravascular hemolysis propensity, defined as follows:

$$\mathrm{CFHI}=\mathrm{HGB}(g/\mathrm{dL})-\frac{\mathrm{HCT}(\%)}{3}$$

Standard automated hematology analyzers measure total Hgb by chemically lysing all red cells in the blood sample, combining intracellular Hgb and any pre-existing cell-free plasma Hgb into a single cumulative value. In contrast, Hct (MCV × RBC) uniquely isolates intact, packed RBC volume. Under baseline physiological homeostasis, Hct is roughly three times the total Hgb concentration (the classic ‘Rule of Three’). CFHI mathematically quantifies the absolute deviation from this physiological encapsulation equilibrium. We hypothesize that a positive shift (CFHI > 0) flags an elevated pool of plasma-free Hgb. Once in circulation, cell-free plasma hemoglobin scavenges endothelial NO, causing smooth muscle contraction and risk of flow disruption.

The last metric, EPVB, was engineered to model the spatial volumetric equilibrium between the central driving erythrocyte column and the peripheral marginated platelet layer, defined as follows:

$$\mathrm{EPVB}=\frac{\mathrm{HCT}(\%)}{\mathrm{PCT}(\%)}$$

While Hct isolates total packed erythrocyte volume driving central axial flow, PCT quantifies the cumulative spatial mass of circulating platelets lining the vessel periphery. Under our model hypothesis, EPVB proxies the physical adequacy of the peripheral fluidic buffer layer. When this ratio shifts markedly toward Hct dominance, the protective peripheral platelet boundary becomes compromised and can no longer shield the vessel wall. Consequently, erythrocytes lose their axial alignment and undergo lateral margination. Without this layer, erythrocytes contact damaged endothelium, increasing friction and impairing flow.

In summary, we synthesized these five core features—the three newly engineered biophysical surrogate indices (EFI, CFHI, and EPVB), alongside baseline MPV and gender—into the BFM. We hypothesize that as this multicellular suspension traverses a stenotic coronary segment, these cellular dimensions dynamically interact with each other and with local stenotic geometry. By governing core-exclusion dynamics, aberrant margination, endothelial mechanical friction, and NO scavenging, these cellular interactions influence trans-lesional pressure drops. Beyond their core mechanical role across a stenosis, these features may also reflect broader vascular health. As circulating blood cells constantly traverse the endovascular system, their structural and quantitative traits could implicitly indicate systemic endothelial wear. Consequently, alongside its primary focus on localized fluid dynamics, the BFM may additionally incorporate background vascular state into its flow estimates.

### 2.3. Statistical Analyses

Statistical analyses used IBM SPSS Statistics (version 26.0). Normality was assessed with the Shapiro–Wilk test. Normally distributed variables were shown as mean ± SD and compared with Student’s *t*-test; non-normal data were shown as median (range) and analyzed using the Mann–Whitney U test. Categorical data were reported as counts and percentages and tested with the chi-square or Fisher’s exact test. Spearman’s rank correlation was used to assess associations between continuous hemorheological parameters and FFR values. A multivariable binary logistic regression model was used to evaluate predictors of coronary ischemia (FFR ≤ 0.80). Continuous predictors were standardized via Z-score for comparison of odds ratios (OR). Adjusted ORs are reported along with their 95% confidence intervals (95% CI). Multicollinearity was checked using Tolerance and VIF, with VIF < 2.5 or Tolerance > 0.40 indicating no collinearity. Model fit was assessed using the Omnibus test, Hosmer–Lemeshow test, and Nagelkerke R^2^. Classifier performance (sensitivity, specificity, accuracy) was evaluated at a 0.50 probability threshold. Receiver operating characteristic (ROC) curves were used to determine the area under the curve (AUC) accompanied by 95% CI. Tests were two-tailed; *p* < 0.05 was significant.

### 2.4. Multimodal NN Fusion

**Figure 2:**
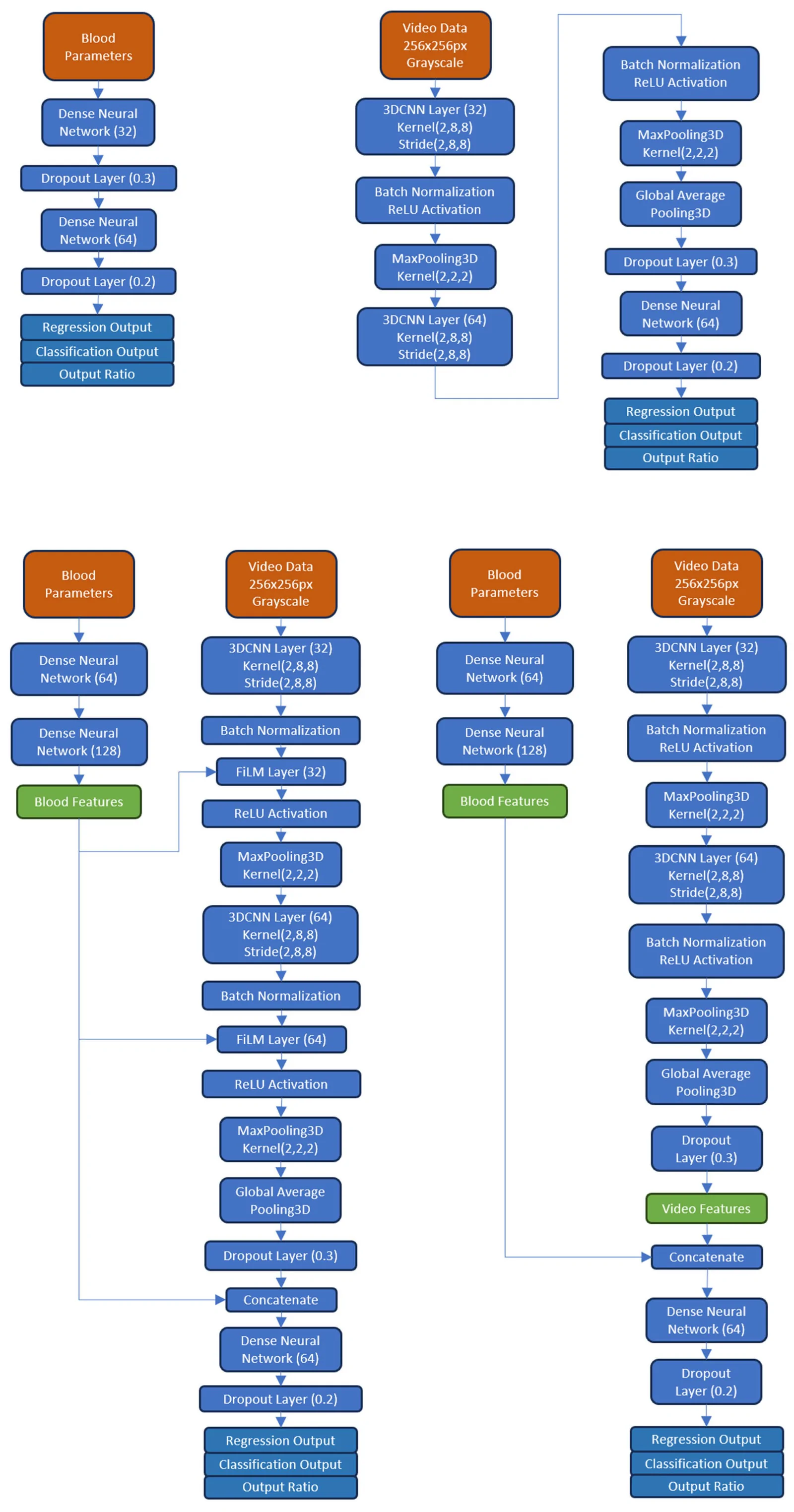
Comparison of network topologies from (**top left**): blood parameters only, (**top right**): video only, (**bottom left**): Multimodal FiLM, (**bottom right**): Multimodal Late Fusion. Orange boxes are inputs, green boxes are intermediate states, and blue boxes are layers. Data flows in the direction of arrows.

This study introduces a multimodal neural network (NN) framework that supplements traditional geometry models by integrating macrovascular features and blood parameters. It uses feature-level conditioning to include non-Newtonian blood dynamics in ischemic risk assessment. We employed a dual-branch neural network with FiLM layers [25], enabling the patient’s hematological profile to modulate the modeling of dynamic ICA video sequences. To ensure a comprehensive input layer, every model configuration that incorporates blood data (Blood Parameters Only, Late Fusion, and FiLM) was formatted to include both raw baseline erythrocyte and thrombocyte indices (RBC, Hgb, Hct, MCV, MCH, MCHC, RDW, PLT, MPV, PCT, PDV and gender) and the derived parameters of the BFM. Four distinct topologies were benchmarked to evaluate individual and combined modal performance: Blood Parameters Only (all parameters vs. BFM), Video Only (angiography videos only), Multimodal Late Fusion (blood parameters and angiography videos), and Multimodal FiLM (angiography video features modulated by blood parameters). Figure 2 shows detailed model compositions.

#### 2.4.1. Dual-Branch Network Topology and Feature-Wise Linear Modulation (FiLM)

The proposed multimodal framework synthesizes heterogeneous data streams through two parallel processing branches: a hematological branch and a deep spatiotemporal video branch.

The Hematological Branch: This stream ingests the full spectrum of the patient’s blood profile, incorporating raw baseline CBC metrics or the BFM parameters. Following per-fold Z-score normalization for numerical stability, these combined features pass through dense layers with dropout regularization. This architecture allows the network to represent both primary cell counts and derived hemorheological setpoints.

The Spatiotemporal Video Branch: This stream digests the standardized 32-frame coordinate-cut (256 × 256 pixels) ICA video sequences. Features are extracted via 3D-CNNs, in which 3D convolution kernels simultaneously downsample the dynamic sequence across both spatial dimensions and temporal frames, effectively preserving contrast agent velocity fields and structural stenosis deformations.

Rather than using a naive late-fusion strategy—which merely concatenates isolated feature vectors before the final prediction layer—our architecture embeds conditioning information directly into the intermediate layers of the image generation pipeline via sequential FiLM layers. Within this framework, the blood features are translated into scaling (γ) and shifting (β) coefficients that operate element-wise on the spatiotemporal feature maps (F) generated by the 3D-CNN layers. The feature-wise linear transformation is mathematically formalized as follows:

(1) *FiLM*(*F_i_*_,_*_c_*| *γ_i_*_,_*_c_*,*β_i_*_,_*_c_*) = *γ_i_*_,_*_c_F_i_*_,_*_c_* + *β_i_*_,_*_c_*

where *F_i_*_,_*_c_* represents the c-th feature map channel for the i-th input sample. Crucially, these conditioning operations are strategically applied immediately prior to the Rectified Linear Unit (ReLU) activation functions. Because the ReLU operator maps negative input values to zero, the sign and magnitude of the tabular-derived γ coefficient can selectively suppress or amplify entire spatial representation channels (γ < 0 systematically forces subsequent ReLU outputs to zero). By using blood features to dynamically gate macrovascular video representations, this architecture transforms the hematological branch from a passive secondary input into an active driver of spatiotemporal attention. Two separate conditioning stages are stacked sequentially: the first stage captures primary spatial edge geometries (luminal borders and catheter boundaries), while the second stage targets high-level, abstract contrast-dispersion patterns. To ensure robust feature propagation, the baseline blood features vector is also concatenated at the final fusion layer, preserving an unmodulated pathway to guide clinical classification.

#### 2.4.2. Tri-Headed Output Optimization and Biophysical Ratio Loss

For all models, the final fused feature representation is mapped to three distinct output heads, each designed to harmonize the mathematical boundary conditions between continuous regression modeling and clinical cutoff thresholds. The network is supervised via three distinct prediction heads:

The Regression Head: Utilizes a Sigmoid activation function to predict the continuous standardized FFR value, optimized via standard Mean Squared Error (MSE).

The Classification Head: Utilizes a Sigmoid activation function to output the binary probability (*p_cls_*) of functional ischemia based on the adjusted diagnostic cutoff.

The Auxiliary Ratio Head: Operates with a linear activation function to predict a composite biophysical score defined as: ScoreRatio = *p_cls_*/(*p_cls_* + *p_reg_* + *ε*), where *p_cls_* is the classification probability, and *p_reg_* is the predicted continuous regression FFR output. Empirically, predictive statistical analyses demonstrated that this combined ratio score correlates significantly more strongly with true, uncompromised FFR measurements than any mathematical head evaluated in isolation. The formula has results between 0 and 1, consistent with other output heads. A very small epsilon value was added to avoid division by zero.

Because clinical FFR operates as a bounded metric, raw FFR values in our dataset ranging between [0.59, 0.97] are mapped from the clinical interval [0.5, 1.0] to a standardized training dynamic range of [0, 1], shifting the standard diagnostic ischemic cutoff threshold of 0.80 to exactly 0.60. To penalize clinically catastrophic errors—such as a continuous regression model predicting a non-ischemic value (>0.80) for a patient whose true physiological state is profoundly ischemic (≤0.80)—a custom Boundary Loss function utilizing Mean Squared Error (MSE) loss (ℒ*_bMSE_*) is integrated:

(2) $$L_{\mathrm{bMSE}}=\mathrm{MSE}(\tilde{y},\hat{y})+0.5\cdot E\left[1[\mathrm{sign}(\tilde{y}-\tau )\neq \mathrm{sign}(\hat{y}-\tau )]\cdot (\tilde{y}-\hat{y}{)}^{2}\right]$$

where τ = 0.60 is the normalized threshold. This directly penalizes clinically consequential regression errors by adding half of the MSE to itself if prediction and real value states contradict. The absolute training objective is governed by a composite loss function (*L*), mathematically formalized as follows:

(3) ℒ = 1.0 ⋅ ℒ*_bMSE_* + 0.3 ⋅ ℒ*_cls_* + 0.2 ⋅ ℒ*_ratio_*

combining continuous regression, value classification, and score ratio. Outputs of the regression head are used as predictions for test metrics, and these predictions are converted to classifications using the ischemic cutoff threshold.

#### 2.4.3. Training Methodology

The performance of our proposed architectures was evaluated through a systematic ablation study, comparing unimodal baselines against multimodal fusion strategies.

To mitigate stochastic dependency between samples from the same patient, we adopted a patient-level splitting strategy. Standard random splitting would inadvertently introduce data leakage, as multiple videos from a single patient would contaminate both the training and validation sets. We employed the StratifiedGroupKFold strategy, ensuring that all videos belonging to the same patient remain within the same fold, regardless of the target label. Patients with mixed FFR labels across their videos (2 of 179) are handled naturally: all videos of the same patient belong to the same fold regardless of label.

Furthermore, a 20% held-out test set reserving approximately 36 patients (≈51 videos) was sequestered prior to any model development, serving as a ‘blind’ benchmark that was never utilized for hyperparameter tuning or early stopping criteria. The remaining 80% of patients formed the training set, of which 20% (16% of all patients) were used for validation. All experiments were conducted using a 5-fold cross-validation strategy across 16 random seeds to account for sampling variance, with final evaluations performed on the test set.

The test set ensures that reported metrics reflect genuine generalization. Patient-level grouping prevents blood parameter leakage that would otherwise inflate performance for the tabular and multimodal models.

The dataset exhibits a class imbalance ratio of approximately 1:1.94 (positive vs. negative). To prevent model bias toward the majority class, we implemented Balanced Batch Sampling, utilizing an oversampling generator that provides an equal number of positive and negative samples per training epoch.

#### 2.4.4. Training Protocol

The training protocol utilized the Adam optimizer (initial LR: 2 × 10^−4^) with an adaptive scheduler that reduces the learning rate with patience of 8 epochs and early stopping with patience of 20 epochs. We chose balanced accuracy (BA) as the monitoring metric, as it is the mean of sensitivity and specificity, is robust to class imbalance, and sensitivity or specificity collapsing gives BA = 0.5. We observed that increasing the training duration to 30 or 50 epochs triggered the scheduler excessively, causing the optimizer to prematurely converge to local minima with limited generalization capacity. A 20-epoch training window, however, maintained a more robust global trajectory.

Furthermore, a batch size of 1 was empirically determined as optimal; larger batch sizes (2, 4, 8, 16) exhibited degraded performance, likely due to reduced gradient update frequency and the sensitivity of internal covariate shift correction in our specific 3D-CNN architecture. Gradient clipping is employed to prevent gradient explosion with batch size 1. Hyperparameters for the models were optimized using Optuna [26] (v4.9.0) with 50 trials, restricted strictly to the inner training/validation folds to prevent any data leakage into the test sets. A define-by-run approach was used, exploring search spaces for learning rates, loss weights, dropout rates, and number of epochs. The detailed search space is given in Appendix A (Table A1). While Optuna provided robust initial guidelines for the core architectural hyperparameters, final training incorporated specific domain adaptations. Rather than a fixed epoch count from the automated search, models were trained up to a maximum ceiling of epochs paired with an adaptive learning rate scheduler (e.g., reducing the learning rate upon validation plateau) to ensure optimal convergence. The task-specific loss balancing weights were further fine-tuned via systematic validation monitoring to balance the gradient contributions of the regression, classification and ratio heads.

Network weights were initialized using standard Keras Glorot (Xavier) uniform initialization. Deterministic weight initialization and data shuffling across folds and random seeds were guaranteed by setting explicit seed controls (tf.random.set_seed and np.random.seed) prior to model instantiation in each fold.

All models were implemented using Python (v3.10.12) with TensorFlow (v2.17.0) and Keras (v3.3.3) frameworks. Model performance and statistical comparisons of modality predictions were conducted using the SciPy (v1.13.1) library. To prevent pseudoreplication across multiple seeds and video runs, we aggregated model predictions into a single ensemble prediction per patient. Continuous predicted FFR values were compared against ground-truth invasive FFR measurements using correlation analyses. Additionally, binary ischemia categorization (≤0.80 threshold) was evaluated using ROC curves, AUC, mean absolute error (MAE), sensitivity, specificity, and balanced accuracy to assess concordance between predicted and clinical ischemia classifications.

To rigorously assess performance differences between the reference model (Multimodal FiLM) and all candidate models, metric-specific paired statistical tests were employed within a patient-level bootstrap framework (1000 iterations). For continuous regression errors (Mean Absolute Error, MAE) and paired metric comparisons, the non-parametric Wilcoxon signed-rank test was applied across the bootstrap resampling distributions. For classification discrimination performance (Area Under the Curve, AUC), patient-level paired bootstrap resampling was utilized to derive empirical *p*-values. To control the family-wise error rate arising from multiple pairwise comparisons against the reference model, Holm-Bonferroni corrections were applied to all resulting *p*-values. We then evaluated the monotonic relationship between predicted values and ground-truth invasive FFR measurements using Spearman’s rank correlation. A *p*-value < 0.05 was considered statistically significant. To evaluate the agreement between predicted continuous FFR values and invasive measurements, Bland–Altman analysis was performed, determining the mean bias and 95% limits of agreement (mean ± 1.96 × SD) across the test set.

In TensorFlow, Keras, and Python, all random processes were made deterministic by defining seed values. At the start of each fold, the fold number is added to the seed, ensuring generator shuffles and weight initialization are independent across folds and model types.

#### 2.4.5. Explainable AI (XAI) via Spatiotemporal Grad-CAM

To overcome the traditional black box limitations, visual interpretability is achieved using Gradient-weighted Class Activation Mapping (Grad-CAM) [27]. Grad-CAM is applied to the first ReLU activation layer of the 3D-CNN pipeline to isolate high-resolution spatial edge features and to map temporal attention variations during contrast agent opacification.

**Figure 3:**
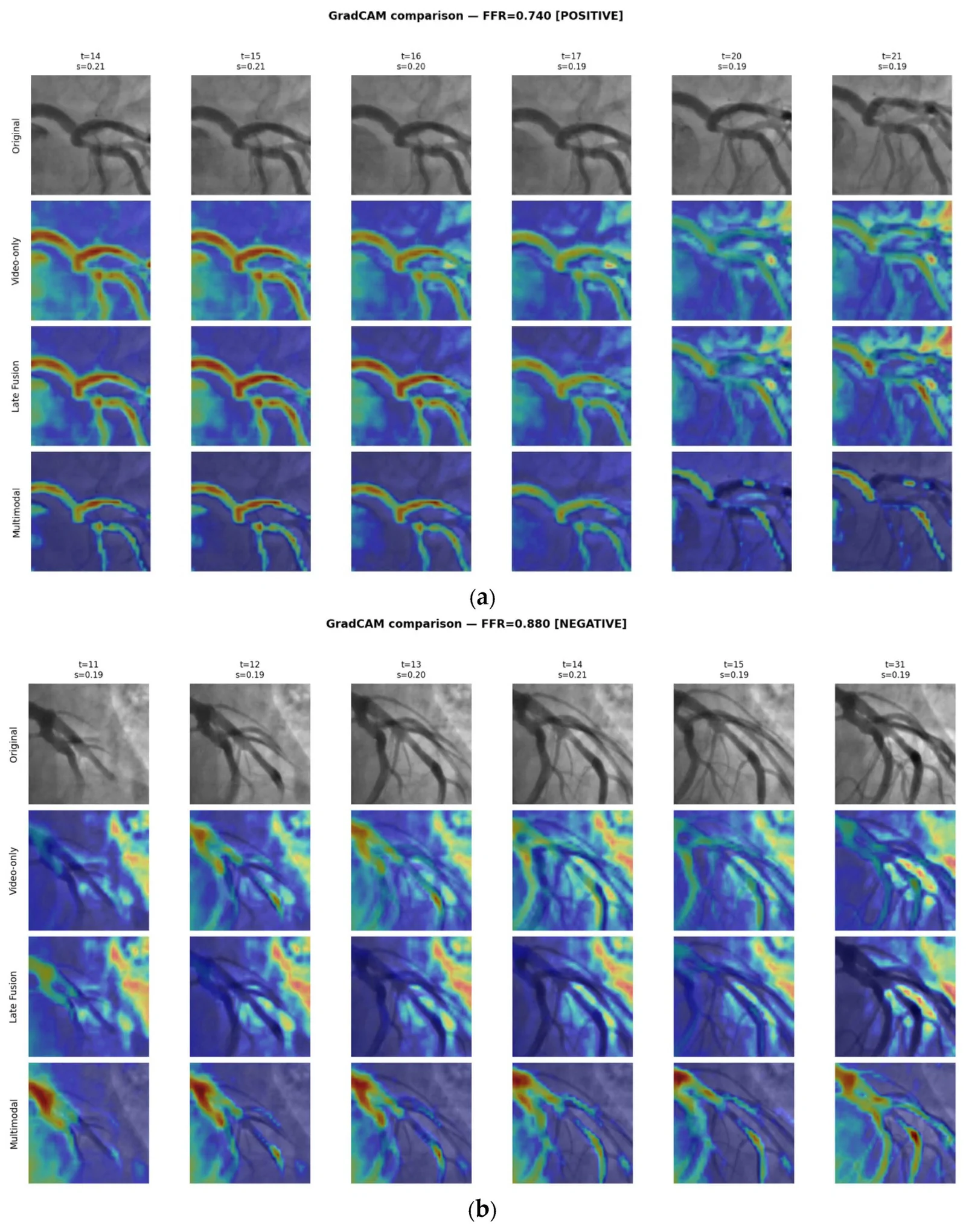
Qualitative Grad-CAM saliency map overlays for representative positive (ischemic) (**a**) and negative (non-ischemic) (**b**) test cases, evaluated at the test fold corresponding to median test performance. Frames are selected based on the highest mean saliency across models. From top to bottom, rows display: raw angiographic frame, video-only baseline, late fusion model, and multimodal FiLM architecture. The color overlay denotes attribution weights ranging from low (blue) to high (red).

For each temporal frame and spatial coordinate, the importance weights for individual feature map channels are computed by applying global average pooling to the gradients of the score head with respect to the feature map activations. These weights are linearly combined with the forward activation maps and then passed through a positive-filtering ReLU operator to eliminate negative gradients that do not contribute to ischemic prediction. The native 32 × 32 spatial saliency maps are subsequently upscaled to the original 256 × 256 pixels using the OpenCV inter-area and bilinear interpolation algorithm. To prevent visual frame-duplication artifacts arising from a temporal stride of 2 in the 3D convolutional blocks, temporal interpolation is performed via precise linear blending between adjacent feature maps. This approach produces a smooth, clear video map of spatial and temporal attention. Interventional cardiologists can use this to confirm that the multimodal network’s focus accurately aligns with both the physical blockage and the flow of blood through the coronary arteries. The representative cases given in Figure 3 were selected based on the test folds and seeds that yielded performance closest to the median error.

## 3. Results

A total of 179 patients undergoing diagnostic ICA and FFR assessment were analyzed (124 males [69.3%], 55 females [30.7%]; mean age: 58.2 ± 10.0 years). Median FFR was 0.84 (range: 0.59–0.97), and functional ischemia (FFR ≤ 0.80) was detected in 60 patients (33.5%). The female cohort was significantly older (64.2 ± 9.4 vs. 55.5 ± 9.2 years, *p* < 0.001), yet male patients demonstrated significantly lower median FFR values (0.83 vs. 0.87, *p* = 0.002) and a higher ischemia prevalence (26.8% vs. 6.7% of the total population; representing 38.7% of males vs. 21.8% of females, *p* = 0.027). All cellular blood parameters and derived hemorheological indices—except PDW—showed significant baseline gender differences (*p* < 0.05), with values generally higher in males except for MPV and EFI (Table 1).

Across the functional ischemic boundary (FFR ≤0.80 vs. >0.80), univariate comparisons did not reach statistical significance. Continuous linear correlation analysis confirmed that MPV was weakly correlated with absolute FFR values (*r_s_* = 0.159, *p* = 0.034). To evaluate the independent predictive capability of classical statistical methods, a multivariable binary logistic regression model was constructed using male gender and Z-score-transformed MPV, EFI, EPVB, and CFHI. The multivariable model demonstrated statistically significant predictive value (*p* = 0.039, Nagelkerke R^2^ = 0.088). Classification was limited by a sensitivity deficit (13.3% vs. 95.8% specificity; accuracy: 68.2%) and an AUC of 0.650 (95% CI: 0.565–0.735, *p* = 0.001). Male gender and EFI showed borderline independent predictive value (Table 2).

The independent test set evaluation (Table 3 and Table 4) provides a patient-level assessment of the models’ generalization capacities using aggregated ensemble predictions, 95% CIs, and pairwise statistical comparisons against the Multimodal FiLM reference. When utilizing BFM parameters (Table 4), the Multimodal FiLM framework achieved the highest point estimates in test AUC (0.621, 95% CI: 0.519–0.713) and balanced accuracy (0.590, 95% CI: 0.505–0.671), whereas the video-only model remained near chance level (AUC 0.482, 95% CI: 0.382–0.585). Furthermore, intermediate feature gating via FiLM significantly outperformed Late Fusion when using all blood parameters (*p* = 0.0025, Table 3), reinforcing that convolutional-stage conditioning is superior to late-stage feature concatenation. However, given the overlap in 95% CIs across certain baseline modalities and non-significant pairwise *p*-values in Table 4, these findings should be interpreted as preliminary.

**Figure 4:**
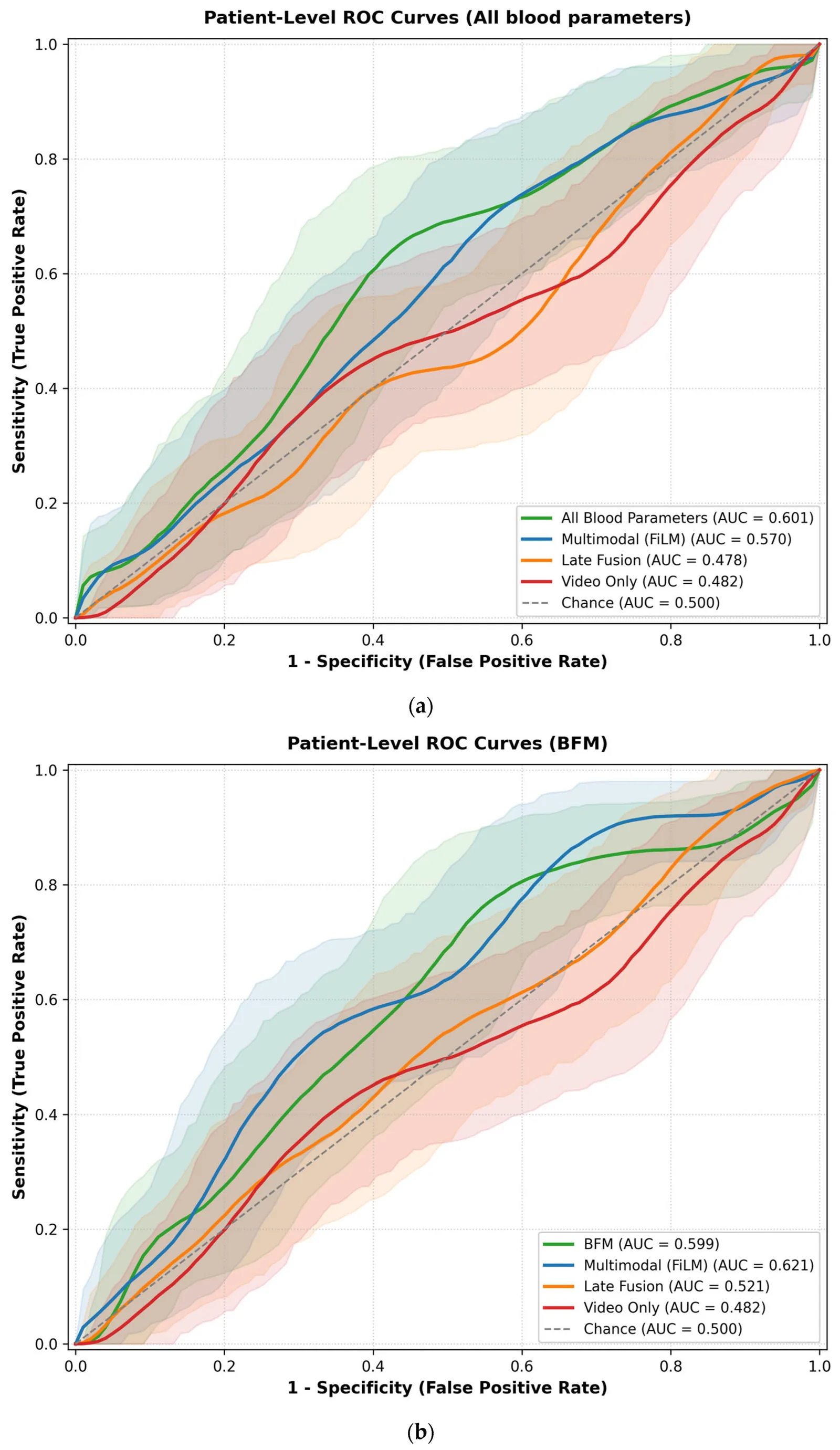
Patient-level ROC Curves for all blood parameters (**a**) and BFM (**b**). Shaded regions represent the 95% confidence intervals. Abbreviations: AUC, area under curve; BFM, blood fluidity model; FiLM, feature-wise linear modulation; FFR, fractional flow reserve; ROC, receiver operating characteristic.

**Figure 5:**
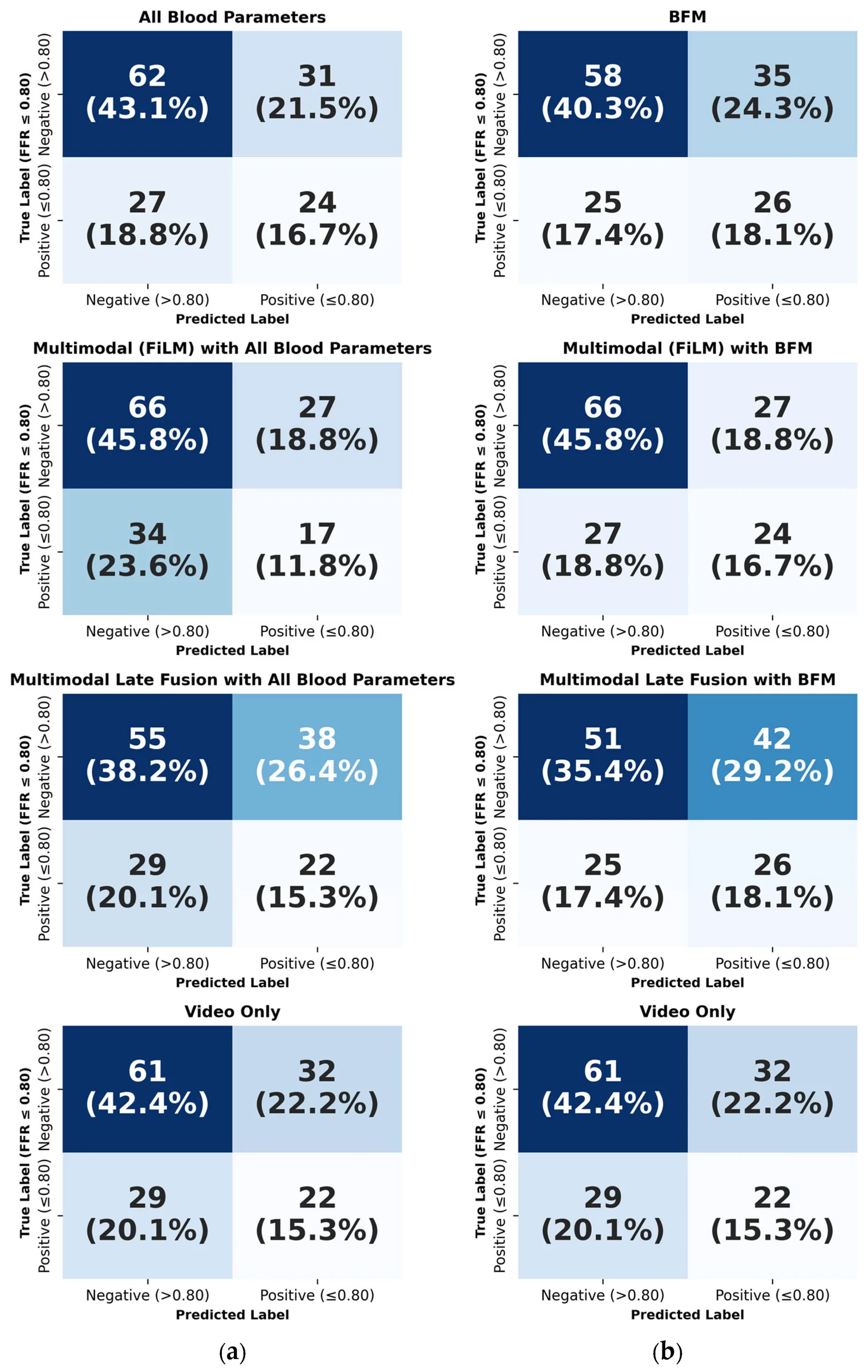
Patient-level confusion matrices evaluated at the binary ischemic threshold (FFR ≤ 0.80) for the test set across modalities for (**a**) all blood parameters and (**b**) BFM. Abbreviations: BFM, blood fluidity model; FiLM, feature-wise linear modulation; FFR, fractional flow reserve.

**Figure 6:**
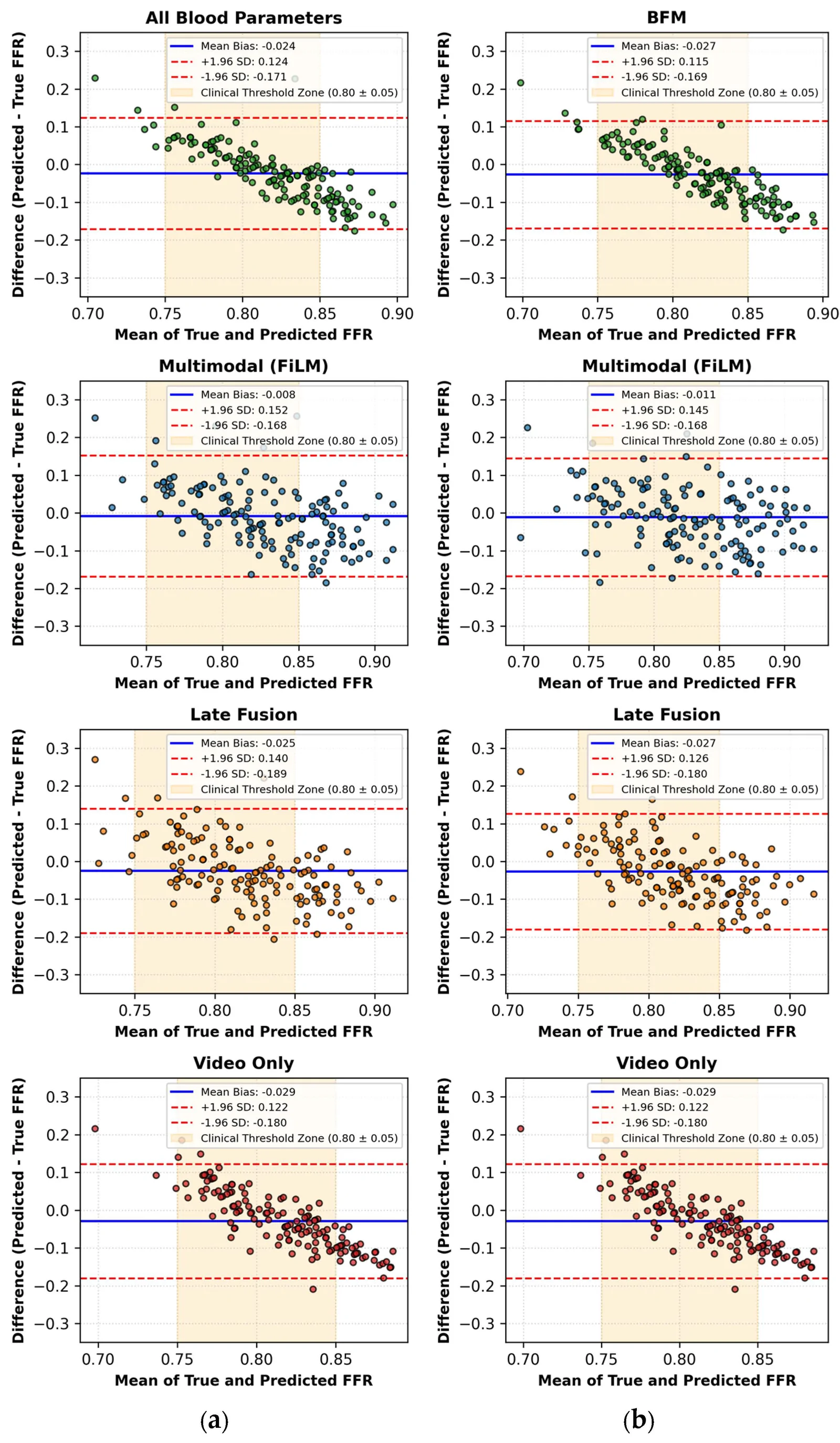
Patient-level Bland–Altman plots evaluated at the binary ischemic threshold (FFR ≤ 0.80) for the test set across modalities for (**a**) all blood parameters and (**b**) BFM. Abbreviations: BFM, blood fluidity model; FiLM, feature-wise linear modulation; FFR, fractional flow reserve.

The comprehensive performance comparison of the evaluated models is visually summarized in Figure 4 and Figure 5. Specifically, Figure 4 presents the ROC curves accompanied by shaded 95% CI regions, illustrating the discrimination capability of each architecture. Figure 5 displays the confusion matrices, detailing the classification trade-offs between true and false predictions across different threshold levels. Bland–Altman plots (Figure 6) illustrate the agreement and error distribution between predicted and measured FFR across the test set. While unimodal baselines and late fusion models demonstrated noticeable proportional bias with sloping error distributions, both multimodal FiLM configurations successfully mitigated this trend, displaying balanced error distributions centered near the zero-difference line. Specifically, the Multimodal FiLM with BFM yielded a mean bias of −0.011 with 95% limits of agreement ranging from −0.168 to 0.145, whereas the Multimodal FiLM with all blood parameters showed a mean bias of −0.008 with limits from −0.168 to 0.152. Within the critical clinical threshold zone (0.80 ± 0.05), both FiLM-conditioned models maintained balanced error spreads without severe directional drift.

To visually verify that model predictions were driven by genuine vascular pathology rather than extraneous background features or image artifacts, Grad-CAM saliency maps were generated across the testing cohort (Figure 3). Qualitative inspection of representative positive and negative cases matching median cohort performance confirmed that all three video architectures focused predominantly on the primary coronary tree during maximum contrast opacification. Notably, FiLM produced more focused spatial activations along the coronary lumen compared to late fusion and video-only baselines, suppressing off-target extravascular noise.

Evaluating the correlation between model predictions and invasive FFR revealed distinct performance patterns across modalities. When incorporating all unrefined blood parameters, none of the configurations reached statistical significance (Table 5). In contrast, utilizing the BFM (Table 6) yielded a marked improvement: the Multimodal FiLM framework achieved the highest and most statistically significant correlation with invasive FFR (*r_s_* = 0.246, *p* = 0.003). The BFM-only model also demonstrated a statistically significant correlation (*r_s_* = 0.193, *p* = 0.021), supporting our hypothesis that CBC-derived BFM parameters carry an independent predictive signal for intracoronary pressure drop. Meanwhile, neither the video-only baseline nor the Multimodal Late Fusion model reached statistical significance (*p* > 0.05).

Finally, a comprehensive quantitative comparison summarizing the discrimination, error, correlation, and statistical significance across all principal architectures is presented in Table 7, highlighting the overall performance advantages of the proposed multimodal integration.

## 4. Discussion

In this study, we show that incorporating patient-specific hemorheological surrogates derived from routine CBC tests improves non-invasive FFR estimation beyond visual angiographic geometry alone. For intermediate coronary stenoses, where macrovascular anatomical severity fails to predict functional ischemia—as reflected by our baseline video-only model (AUC: 0.482)—our findings confirm that trans-stenotic pressure drops are also governed by the cellular dynamics of blood. By unifying dynamic spatiotemporal features from ICA sequences with an engineered BFM via FiLM modulation, our multimodal framework achieved a diagnostic AUC of 0.621, outperforming both pure visual geometry and unrefined full-blood panels. While these diagnostic gains remain modest, they highlight a practical, zero-additional-cost approach that leverages routine, readily available laboratory data to capture the biophysical complexity of coronary flow.

To evaluate the functional necessity of our multimodal framework, we first assessed the empirical performance of unimodal visual baselines. The video-only baseline failed critically, yielding a test AUC of 0.482. This shows that purely visual representations from standard ICA projections are insufficient to predict functional flow. Conventional computer vision architectures detect macrovascular narrowing but implicitly treat the coronary arterial tree as rigid conduits, neglecting systemic fluid resistance, vessel compliance, microvascular friction, and the dynamic rheology of blood [3]. This limitation recurs throughout the literature.

Cho et al. [14] achieved 82% diagnostic accuracy only after manually supplementing angiographic features with conventional clinical and demographic variables (age, gender, body surface area, and involved segment), confirming that raw images benefit from physiological context. While their approach incorporates valuable demographic and macro-anatomical information, our framework seeks to complement visual geometry from a different physiological angle. By integrating routine CBC-derived metrics via the BFM, we aim to explore the potential role of blood fluidity and intra-luminal fluid dynamics, offering a preliminary hemorheological perspective alongside angiographic data.

Similarly, Mahendiran et al. [28] achieved an AUC of 0.81 using a ResNet18 model on angiogram patches, yet remained constrained by the rigid-vessel assumption. The framework by Zimmerman et al. [29] plateaued near 80% accuracy, indicating that isolated visual or pressure signals cannot fully capture hyperemic flow dynamics. To process raw single-view angiographic video sequences without heavy proprietary processing, we selected a 3D-CNN as an efficient foundation model for spatiotemporal feature extraction. Notably, dataset constraints limited our model’s performance: in our modest cohort, a single misclassification shifted the overall AUC by 2 percentage points, and target FFR values were centered around the 0.80 cutoff, increasing learning difficulty near the decision boundary. In contrast, Mineo et al. [5] achieved a peak AUC of 0.95 using a pretrained 3D-CNN coupled with a spatiotemporal transformer; however, their framework used a dataset three times larger and had significantly higher model parameter capacity.

With hematological context established, the key architectural question became how to optimally fuse these dynamic vectors with angiographic features. The Late Fusion model (mean test AUC = 0.521) substantially underperformed FiLM conditioning with BFM (mean test AUC = 0.621). This discrepancy aligns with architectural theory: Late Fusion merely concatenates isolated representations at the decision layer, whereas FiLM dynamically applies channel-wise affine scaling (γ) and shifting (β) to intermediate convolutional feature maps. This mechanism allows the blood vector to actively modulate how the 3D-CNN extracts spatiotemporal features at every stage. By predicting only two scalar parameters per feature channel, FiLM incurs minimal computational overhead compared to multi-head self-attention matrices (Query, Key, Value), providing a lightweight design that effectively prevents overfitting in smaller cohorts. Crucially, as our modality comparisons show, conditioning visual maps with unrefined, high-dimensional blood panels introduced cross-modal noise that degraded FiLM performance (AUC: 0.570), whereas the domain-engineered BFM provided a condensed biophysical fingerprint that interacted synergistically with geometry (AUC: 0.621). To bridge continuous FFR value estimation with binary ischemia classification near decision-critical thresholds, we implemented a composite objective function that aligns regression outputs with classification probabilities. This enforced a clinical prior that minimizes diagnostic ambiguity near the 0.80 FFR boundary. Beyond threshold optimization, the agreement analysis provides fundamental insights into how cross-modal conditioning reshapes error dynamics. While unimodal visual baselines and late-fusion structures exhibited a pronounced proportional bias—systematically overestimating severe lesions and underestimating non-ischemic cases—the integration of hemorheological conditioning via FiLM effectively eliminated this directional drift. By smoothing out systematic prediction bias across the continuous FFR spectrum, the BFM-FiLM framework achieves a balanced error profile, particularly within the diagnostic grey zone (0.80 ± 0.05). Nevertheless, the observed individual-level error dispersion highlights the multi-factorial nature of coronary physiology, reinforcing that such non-invasive AI platforms are best suited to function as supplementary clinical decision-support tools rather than autonomous diagnostic arbiters. Qualitative saliency heatmaps further supported this internal consistency by visually pointing toward coronary arterial segments rather than background artifacts. However, as quantitative spatial alignment could not be computed due to the lack of ground-truth vessel segmentation masks, these visual findings should be interpreted as exploratory visual evidence of model focus.

Perhaps the most compelling insight from our study is that systemic blood biophysics carries a distinct, quantifiable diagnostic signal for functional coronary ischemia—revealing an independent biological dimension completely invisible to visual geometry alone. Across both classical statistical and machine learning paradigms, blood metrics showed predictive value: multivariable logistic regression of BFM parameters yielded an AUC of 0.650 (*p* = 0.001, specificity: 95.8%), while standalone blood classifiers achieved test AUCs of 0.601 (all blood parameters) and 0.599 (BFM). Crucially, our comparative multimodal analyses show that unrefined all-blood parameters introduce feature noise when conditioned with visual geometry, as integrating them with video features via FiLM degraded performance and dropped the AUC to 0.570. In contrast, although our BFM yielded a similar baseline AUC of 0.599, its biophysical representation interacted synergistically with 3D spatiotemporal video features under FiLM modulation, elevating multimodal performance to an AUC of 0.621. This confirms that while general blood parameters carry an independent biological signal, the engineered BFM optimizes cross-modal feature interaction, effectively complementing lumen geometry to capture localized coronary ischemia. The better performance of FiLM conditioning with the BFM highlights the advantage of biophysical feature abstraction when integrating multimodal clinical data, especially in a modest cohort. The BFM overcomes this bottleneck by consolidating the non-Newtonian cellular biophysics of stenotic coronary flow into composite indices [12,13,17]. Within stenotic segments, erythrocyte deformability and axial laminar dynamics largely govern blood flow [17]. When deformability is impaired—as indicated by elevated MCHC (cytoplasmic rigidity) [30] and increased RDW (size-driven microscale turbulence) [31]—rigid erythrocytes fail to remain axial and marginate near vessel walls, escalating vascular resistance and shear stress fluctuations [12]. These flow disruptions even facilitate plaque formation, with RDW serving as a key indicator of vascular risk through its impact on blood flow [31]. Our EFI (RDW × MCHC) effectively integrates these dual red cell mechanisms. Furthermore, extreme trans-stenotic shear forces can induce mechanical microhemolysis [13], yielding cell-free hemoglobin that our CFHI captures as a surrogate for local shear trauma and NO depletion-mediated vasoconstriction. Extending beyond isolated RBCs, the EPVB and MPV capture critical intercellular interactions across the lesion, where marginated platelets maintain endothelial barrier integrity [32,33] and actively clear rigid or senescent erythrocytes [34]. Intriguingly, while elevated MPV is conventionally interpreted as an adverse marker of inflammatory platelet activation in acute coronary events [35], higher MPV in our cohort was weakly correlated with higher FFR (*r_s_* = 0.159, *p* = 0.034), reflecting FFR preservation. This finding is best understood in light of our cohort characteristics: all patients presented with established CAD and comparable stenosis severity under baseline diagnostic conditions. In this non-acute setting, larger, metabolically active platelets appear to reflect functional endothelial barrier defense and vascular stability rather than an active thrombotic state. Another concern with blood parameters is their fluctuating nature. Because we excluded white blood cells for this reason, recent evidence shows that routine CBC metrics cluster around highly stable, individualized physiological setpoints over long periods, acting as a conserved biological blueprint rather than a transient snapshot [36]. This provides a strong physiological rationale for using pre-procedural hematological vectors and excluding chronological age, since these high-dimensional cellular profiles directly encode systemic biological aging and vascular inflammation [37].

This study has several limitations that warrant consideration regarding the generalizability and clinical relevance of the proposed methodology. First, we acknowledge the limitation of generalizability due to our single-center retrospective cohort and emphasize the critical need for external validation across diverse multi-center populations and varied angiographic platforms. Furthermore, this single-center design resulted in relatively small per-fold validation sets that introduce sampling variance, where a single misclassification can noticeably alter fold-level metrics; thus, prospective multi-center validation across varied angiographic platforms is required. Second, to maintain structural baseline consistency, we restricted the evaluation to single-stenosis LAD lesions using single-view 2D ICA projections. This omits complex multi-vessel disease, bifurcations, and 3D luminal reconstructions that mitigate foreshortening or vessel-overlap artifacts, thereby constraining the direct extrapolation of our findings to real-world, anatomically complex populations.

Third, our model provides only a single, lesion-level distal FFR estimate and cannot evaluate the longitudinal distribution of pressure loss along the target vessel. As highlighted in contemporary invasive coronary physiology, pressure pullback analysis using indices such as the Pullback Pressure Gradient (PPG) is essential to discriminate focal from diffuse patterns of CAD and guide targeted revascularization strategies [38]. Because our framework relies on cropped angiographic videos trained against a single distal FFR value, it cannot incorporate full-vessel imaging and spatially registered pressure-pullback data, a key physiological limitation.

Fourth, while our engineered five-parameter BFM leverages routine CBC metrics to capture non-Newtonian flow dynamics, these indices serve as biophysical surrogates rather than direct rheological measurements. Fifth, while Grad-CAM visual evaluations provide plausible spatial heatmaps aligning with coronary arteries, these observations remain strictly qualitative. Due to the absence of manual ground-truth vessel segmentation masks in our dataset, precise quantitative spatial overlap metrics (such as Dice similarity coefficients or intersection-over-union) could not be computed. Consequently, saliency map interpretations should be regarded as exploratory visual support rather than definitive anatomical mapping. Finally, this retrospective dataset lacked direct physiological metrics of microvascular resistance, limiting the ability to further calibrate downstream microcirculatory dynamics.

To address these limitations, future research could explore two complementary avenues. First, to broaden diagnostic scope, the multimodal AI framework could advance from single-point distal FFR estimation to full-vessel, 3D multi-view reconstructions integrated with spatially registered pressure-pullback profiles. Second, while the present model relies on routine CBC metrics as biophysical surrogates for cellular components, expanding the feature space to include broader plasma-phase dynamics alongside direct rheological measurements may offer a more holistic representation of intravascular fluid dynamics and microvascular interactions.

## 5. Conclusions

In intermediate CAD, non-invasive FFR estimation remains a substantial challenge, as anatomical stenosis severity alone does not always directly correlate with functional ischemia. While current image-based computational methods primarily assess macrovascular geometry, hemorheological factors may also influence blood flow dynamics. To incorporate non-Newtonian blood properties into functional assessment without requiring direct rheological testing, we used routine CBC metrics to formulate a simplified BFM as a practical surrogate. Using a multimodal NN framework with FiLM, we structurally coupled dynamic spatiotemporal angiographic sequences with these hematological vectors, modulating visual feature extraction across convolutional stages.

This study evaluated a single-center retrospective dataset and demonstrated modest classification sensitivity. Nonetheless, the findings show that integrating blood biophysical characteristics with vessel geometry within a single computational framework is feasible. By framing coronary ischemia as an integrated outcome, these findings provide a constructive basis for future refinements in non-invasive ischemia prediction.

## Figures and Tables

**Table 1:** Baseline demographic, clinical, and comprehensive hemorheological profiles of the study population, stratified by gender, functional ischemia status, and continuous FFR correlations.

	Gender			Ischemia			FFR Correlation	
Model	Male (*n* = 124, 69.3%)	Female (*n* = 55, 30.7%)	*p* Value	FFR ≤ 0.80	FFR > 0.80	*p* Value	*r_s_*	*p* Value
Age (years, mean ± SD)	55.5 ± 9.2	64.2 ± 9.4	<0.001	57.9 ± 7.6	58.3 ± 11.1	0.830	0.035	0.646
HGB (g/dL, mean ± SD)	14.6 ± 1.6	12.7 ± 1.3	<0.001	14.1 ± 1.7	14 ± 1.8	0.581	−0.060	0.424
HCT (%, median/mean ± SD)	44.5 (27.8–54.2)	39.3 (25.6–50)	<0.001	42.6 ± 5	42.4 ± 5	0.909	−0.020	0.789
RBC (×10^12^/L, mean ± SD/median)	5.1 ± 0.5	4.6 ± 0.6	<0.001	4.9 (3.1–6)	4.9 (2.9–6.8)	0.773	−0.014	0.847
MCV (fL, median)	88.1 (64.7–98.8)	86.3 (60.3–110)	0.016	87.8 (60.3–110)	87.4 (63.1–98.8)	0.800	0.024	0.753
MCH (pg, median)	29.4 (20.9–33.2)	28.3 (19.8–40.2)	<0.001	29.2 (19.8–40.2)	29.2 (20.1–33.2)	0.713	0.063	0.403
MCHC (g/dL, median)	33.3 (28.7–36.6)	32.7 (29.8–35.1)	0.001	33.1 (30.4–36.6)	33.1 (28.7–36.1)	0.354	−0.100	0.183
RDW (%, median)	13.6 (12–16.7)	14.3 (11.8–19.3)	0.001	14 (12.6–16.7)	13.7 (11.8–19.3)	0.376	−0.011	0.883
PLT (×10^9^/L, median)	247.5 (70–484)	285 (134–579)	0.006	252 (70–579)	265 (112–486)	0.580	0.042	0.580
MPV (fL, median)	8.2 (6.5–11.6)	8.5 (6.7–13.2)	0.034	8.1 (6.5–11.5)	8.3 (6.6–13.2)	0.066	0.159	0.034
PCT (%, median)	0.20 (0.06–0.38)	0.25 (0.14–0.57)	<0.001	0.20 (0.06–0.57)	0.22 (0.10–0.38)	0.085	0.137	0.067
PDW (%, mean ± SD)	51.8 ± 8.6	51.1 ± 9.1	0.601	52.6 ± 7.7	51.1 ± 9.6	0.263	0.004	0.958
EFI (median)	454 (396–556)	467 (388–580)	0.048	467 (417–556)	453 (388–581)	0.074	−0.082	0.276
CFHI (median)	−0.33 (−1.77–1.6)	−0.23 (−1.47–0.7)	0.002	−0.1 (−1.77–1.3)	−0.13 (−1.2–1.6)	0.375	−0.109	0.146
EPVB (median)	218 (110–608)	158 (73–294)	<0.001	218 (73–608)	187 (90–413)	0.101	−0.137	0.068
FFR (median)	0.83 (0.59–0.96)	0.87 (0.66–0.97)	0.002	0.76 (0.59–0.80)	0.87 (0.81–0.97)	<0.001		
Positive FFR (n, %)	48, 26.8%	12, 6.7%	0.027					

Abbreviations: CFHI, cell-free hemoglobin index; dL, deciliter; EFI, erythrocyte fragility index; EPVB, erythrocyte platelet volume balance; FFR, fractional flow reserve; fL, femtoliter; g, gram; HCT, hematocrit; HGB, hemoglobin; L, liter; MCV, mean corpuscular volume; MCH, mean corpuscular hemoglobin; MCHC, mean corpuscular hemoglobin concentration; MPV, mean platelet volume; PCT, plateletcrit; PDW, platelet distribution width; pg, picogram; PLT: Platelet count; RBC, red blood cells; RDW, red cell distribution width; rs, Spearman’s rank correlation coefficient; SD, standard deviation.

**Table 2:** Bivariate and Multivariable Binary Logistic Regression Analysis for Predicting Functional Coronary Ischemia.

Variable	Bivariate OR (95% CI)	*p*-Value	Multivariable Adjusted OR (95% CI)	*p*-Value
Male Gender (vs. Female ref.)	2.263 (1.085–4.719)	0.029	2.253 (0.974–5.210)	0.058
MPV (per 1 SD increase)	0.736 (0.523–1.036)	0.079	0.821 (0.579–1.164)	0.269
EFI (per 1 SD increase)	1.310 (0.960–1.787)	0.089	1.384 (0.992–1.930)	0.056
EPVB (per 1 SD increase)	1.252 (0.920–1.704)	0.152	1.072 (0.757–1.518)	0.696
CFHI (per 1 SD increase)	1.245 (0.905–1.714)	0.178	1.072 (0.760–1.513)	0.692

Abbreviations: OR, odds ratio; CI, confidence interval; MPV, mean platelet volume; EFI, erythrocyte fragility index; EPVB, erythrocyte-platelet volume balance; CFHI, cellular free hemoglobin index; SD, standard deviation. Continuous parameters were Z-score transformed prior to modeling. Multivariable Model Summary: Overall Model *p* = 0.039; Nagelkerke R^2^ = 0.088; Hosmer–Lemeshow test *p* = 0.227. All VIF values were <1.30, confirming no multicollinearity.

**Table 3:** Comparison of test set modality performance with all blood parameters.

Model	All Blood Parameters (95% CI)	Multimodal FiLM (95% CI)	Multimodal Late Fusion (95% CI)	Video Only (95% CI)
AUC	0.601 (0.504–0.691)	0.570 (0.466–0.665)	0.478 (0.375–0.581)	0.482 (0.382–0.585)
*p*-value vs. FiLM (AUC)	0.436	Reference	0.102	0.436
MAE	0.063 (0.056–0.071)	0.065 (0.057–0.074)	0.072 (0.064–0.081)	0.068 (0.061–0.076)
*p*-value vs. FiLM	0.491	Reference	0.008	0.311
Sensitivity	0.471 (0.333–0.600)	0.333 (0.197–0.460)	0.431 (0.293–0.575)	0.431 (0.286–0.569)
Specificity	0.667 (0.567–0.760)	0.710 (0.610–0.802)	0.591 (0.484–0.687)	0.656 (0.565–0.756)
BA	0.569 (0.487–0.649)	0.522 (0.438–0.602)	0.511 (0.421–0.602)	0.544 (0.461–0.625)

Abbreviations: AUC, area under curve; BA, balanced accuracy; CI, confidence interval; FiLM, feature-wise linear modulation; MAE, mean absolute error.

**Table 4:** Comparison of Test Set Modality Performance with BFM.

Model	BFM (95% CI)	Multimodal FiLM (95% CI)	Multimodal Late Fusion (95% CI)	Video Only (95% CI)
AUC	0.599 (0.503–0.693)	0.621 (0.519–0.713)	0.521 (0.410–0.623)	0.482 (0.382–0.585)
*p*-value vs. FiLM (AUC)	0.558	Reference	0.060	0.092
MAE	0.064 (0.058–0.071)	0.065 (0.058–0.073)	0.068 (0.061–0.076)	0.068 (0.061–0.076)
*p*-value vs. FiLM (MAE)	0.959	Reference	0.916	0.916
Sensitivity	0.510 (0.370–0.636)	0.471 (0.341–0.605)	0.510 (0.370–0.655)	0.431 (0.286–0.569)
Specificity	0.624 (0.526–0.723)	0.710 (0.614–0.800)	0.548 (0.452–0.650)	0.656 (0.565–0.756)
BA	0.567 (0.474–0.651)	0.590 (0.505–0.671)	0.529 (0.440–0.620)	0.544 (0.461–0.625)

Abbreviations: AUC, area under curve; BA, balanced accuracy; CI, confidence interval; BFM, blood fluidity model; FiLM, feature-wise linear modulation; MAE, mean absolute error.

**Table 5:** *r* and *p* values of Spearman’s correlation for modality predictions with all blood parameters.

Modality	Correlation Coefficient *r_s_*	Statistical Significance *p*-Value
All blood parameters	0.119	0.154
Video only	−0.040	0.635
Multimodal Late Fusion	0.006	0.940
Multimodal FiLM	0.113	0.177

Abbreviations: FiLM, feature-wise linear modulation; *r_s_*, Spearman’s rank correlation coefficient.

**Table 6:** *r* and *p* values of Spearman’s correlation for modality predictions with BFM.

Modality	Correlation Coefficient *r_s_*	Statistical Significance *p*-Value
BFM	0.193	0.021
Video only	−0.040	0.635
Multimodal Late Fusion	0.111	0.186
Multimodal FiLM	0.246	0.003

Abbreviations: BFM, blood fluidity model; FiLM, feature-wise linear modulation; *r_s_*, Spearman’s rank correlation coefficient.

**Table 7:** Concise summary comparing the proposed multimodal BFM-FiLM framework against key baseline architectures on the independent test set.

Performance Metric	Video Only (Baseline)	Multimodal Late Fusion (BFM)	Multimodal FiLM + BFM (Proposed)	All-Blood FiLM (Baseline)
Test AUC	0.482	0.521	0.621	0.570
Balanced Accuracy (BA)	0.544	0.529	0.590	0.522
MAE	0.068	0.068	0.065	0.065
Spearman Correlation (*r_s_*) (*p*-value)	−0.040 (*p* = 0.305)	0.111 (*p* = 0.320)	0.246 (Reference)	0.113 (*p* = 0.491)

Abbreviations: AUC, area under curve; BA, balanced accuracy; BFM, blood fluidity model; FiLM, feature-wise linear modulation; MAE, mean absolute error; *r_s_*, Spearman’s rank correlation coefficient.

## Data Availability

The data presented in this study are available on request from the corresponding author due to ethical reasons.

## Abbreviations

The following abbreviations are used in this manuscript:

AI	Artificial intelligence
AUC	Area under curve
BA	Balanced accuracy
BFM	Blood fluidity model
CAD	Coronary artery disease
CBC	Complete blood count
CFD	Computational fluid dynamics
CFHI	Cell-free hemoglobin index
CI	Confidence interval
CNN	Convolutional neural network
EFI	Erythrocyte fragility index
EPVB	Erythrocyte platelet volume balance
FFR	Fractional flow reserve
FiLM	Feature-wise linear modulation
Hct	Hematocrit
Hgb	Hemoglobin
ICA	Invasive coronary angiography
LAD	Left anterior descending
LR	Learning rate
MAE	Mean absolute error
MCH	Mean corpuscular hemoglobin
MCHC	Mean corpuscular hemoglobin concentration
MCV	Mean corpuscular volume
MPV	Mean platelet volume
MSE	Mean squared error
NN	Neural network
NO	Nitrous oxide
OR	Odds ratio
PCT	Plateletcrit
PDW	Platelet distribution width
PINN	Physics-informed neural network
PLT	Platelet count
PPG	Pullback pressure gradient
QFR	Quantitative flow ratio
RBC	Red blood cell count
RDW	Red blood cell distribution width
ReLU	Rectified linear unit
ROC	Receiver operating characteristic
ROI	Region of interest
SD	Standard deviation
VIF	Variance inflation factor
WBV	Whole blood viscosity

## Appendix A

Hyperparameter exploration was conducted using Optuna with 50 trials restricted strictly to the inner training/validation folds to prevent any data leakage into the test sets. The search space explored during Optuna trials is given in Table A1.

While Optuna provided robust initial guidelines for the core architectural hyperparameters, final training incorporated specific domain adaptations. Rather than a fixed epoch count from the automated search, models were trained up to a maximum ceiling of epochs paired with an adaptive learning rate scheduler (e.g., reducing the learning rate upon validation plateau) to ensure optimal convergence. The task-specific loss balancing weights (L_bMSE_, L_cls_, L_ratio_) were further fine-tuned via systematic validation monitoring to balance the gradient contributions of the regression and classification heads.

**Table A1:** Hyperparameter exploration.

Hyperparameter	Search Strategy	Search Space/Range	Value Selected
Learning Rate	Log-Uniform	[1 × 10^−5^, 5 × 10^−3^]	2 × 10^−4^ (adaptive)
Max Epochs	Uniform Int	[30,200]	20
Regression Weight	Uniform Float	[0.05,2.0]	1
Classification Weight	Uniform Float	[0.05,2.0]	0.3
Ratio Weight	Uniform Float	[0.05,0.5]	0.2
Dropout 1	Uniform Float	[0.10,0.50]	0.3
Dropout 2	Uniform Float	[0.05,0.40]	0.2
